# Akt inhibitors: mechanism of action and implications for anticancer therapeutics

**DOI:** 10.1186/1750-9378-8-49

**Published:** 2013-12-13

**Authors:** Jaikrit Bhutani, Asfandyar Sheikh, Asfandyar Khan Niazi

**Affiliations:** 1 Post Graduate Institute of Medical Sciences, Rohtak, Haryana, India; 2 Dow Medical College, Dow University of Health Sciences, Karachi, Pakistan; 3 Shifa College of Medicine, H-8/4, Islamabad, Pakistan; 4 Pakistan Research Evolution Scientific Society, Baba-e-Urdu Road, Karachi 74200, Pakistan

## Abstract

Akt, better known as protein kinase B (PKB), is a serine/threonine-specific protein kinase which acts as mediator via PI3K/Akt pathway in many biological processes like glucose metabolism, apoptosis, cell differentiation and transcription. Akt1 gene amplification has been implicated in gastric carcinoma while Akt2 amplification has been linked with ovarian, pancreas, breast and stomach tumors. The use of Akt inhibitors as monotherapy or in combination with other anticancer drugs could be useful for combating drug resistance and improving response. Thus, comprehensive understanding of Akt and its linked signaling pathways (PI3K, PKB, mTOR etc.) is necessary to lead to newer drug development and use.

## Letter to the editor

Akt, better known as protein kinase B (PKB), is a serine/threonine-specific protein kinase which acts as mediator in many biological processes like glucose metabolism, apoptosis, cell differentiation and transcription. Three members in the Akt family have been identified until now, namely Akt1, Akt2 and Akt3. While Akt2 is mostly involved in glucose transport and Akt3 is highly expressed in brain tissue, Akt1 plays a key role cellular survival and metabolism [1,2].

**Figure 1:**
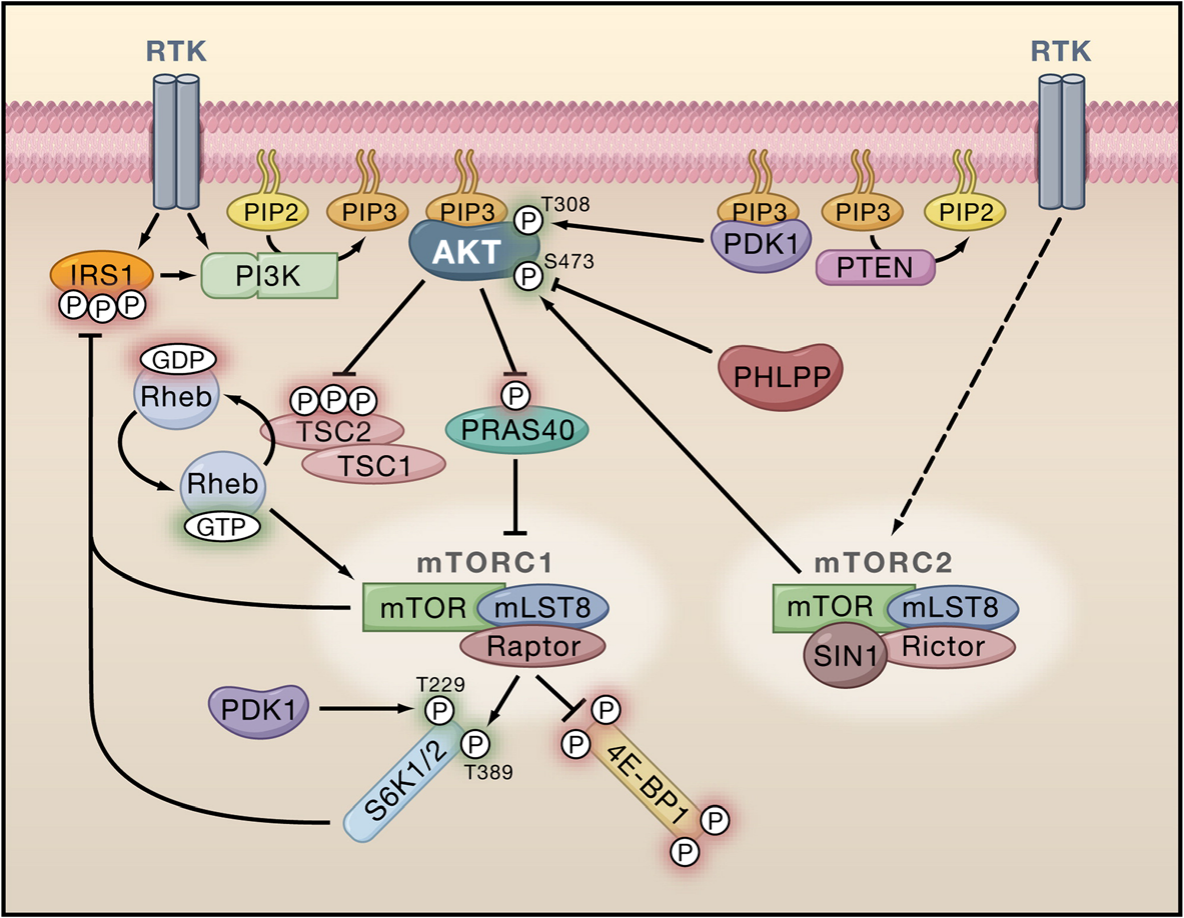
**The PI3K/Akt/mTOR signaling pathway [**[3]**].**

The Akt cascade is activated by a host of events, most commonly via binding of ligands such as growth factors, cytokines and hormones to various receptors, the most important of which are receptor tyrosine kinases (RTK) (Figure 1). Binding of ligands to RTK causes autophosphorylation of tyrosine residues on the intracellular domain of the receptor. This causes the recruitment of PI3K to the phosphotyrosine residues via SH2 domain adapters in the regulatory domain (p85) of PI3K. This causes conformational changes in the catalytic domain of PI3K, which in turn results in kinase activation. This is followed by the PI3K mediated phosphorylation of membrane bound PIP2 to generate PIP3. PIP3 then binds to the PH domain of Akt, thereby anchoring it to the plasma membrane and allowing its phosphorylation and activation by PDK1 [3]. The activity of Akt is negatively regulated by PTEN, SHIP and CTMP [4].

The mechanisms of involvement of the Akt pathway in tumorigenesis are multifold. Activated Akt has well- established anti-apoptotic activities. These are carried via inhibition of release of cytochrome c from the mitochondria or by its regulatory effect on various downstream effectors, e.g. NF-κB, Bcl-2 family proteins, FOXO transcription factors and MDM2, which in turn stimulate tissue growth [5,6]. In addition, Akt activation mediates cell cycle progression via inhibition of glycogen synthase kinase 3beta, opposing the action of p21WAF1 and p27Kip1 and by phosphorylation of AKT/mTOR kinases [7]. The latter results in increased translation of cyclin D1, D3, and E transcripts and carries special significance with regard to anti-cancer therapeutics. mTOR inhibition by rapamycin derivative, everolimus, has been shown by Majumder et al. to reverse AKT-dependent prostate intraepithelial neoplasia [8].

The Akt signaling pathway also influences other facets of tumorigenesis. For example, Akt stimulates angiogenesis facilitating tumor growth [9]. This is due to the mediation of the effects of VEGF by the Flk1/VEGFR2-PI3K-AKT pathway [10]. Other hallmarks of malignancy such as tumor invasion and metastasis are also affected by Akt activation [11]. This is due to the increased secretion of matrix metalloproteinases and the induction of epithelial–mesenchymal transition [12]. Akt has also been shown to stimulate telomerase activity and replication [13].

The spectrum of mutations leading to Akt activation is diverse and mostly affects the PI3K/Akt/mTOR pathway (Table 1) [14]. Amplication and overexpression of Akt2 was first shown to occur in ovarian cancers [15]. Since then, overexpression of Akt2 has been shown to occur in pancreatic cancers, hepatocellular carcinomas, colorectal cancers, stomach cancers and various forms of breast cancers [16-19]. On the other hand, amplification of Akt1 is relatively uncommon, and has been detected in only a few cases of gastric carcinoma and gliosarcoma [20,21]. Expression of Akt3 mRNA has also been found to be upregulated in estrogen receptor-negative breast carcinomas [22]. Furthermore, Akt has also been found as a culprit for tumor cell resistance to chemotherapy while treating breast cancer, ovarian cancer and pancreatic cancer [16,23]. A mutation in the Akt1 gene is also known to cause the Proteus Syndrome [24]. Akt1, measured by immunohistochemistry techniques, has been shown to be a marker of response to radiation therapy in head and neck cancers [25].

**Table 1:** **Common mutations in the PI3K/Akt/mTOR pathway [**[14]**]**

**Targets**	**Genetic alteration**	**Cancer type**
PIK3CA (phosphoinositide-3-kinase, catalytic, *α*-polypeptide)	Mutations	Breast, endometrial, colon, upper digestive tract, gastric, pancreas, ovarian, liver, brain, oesophageal, lung, melanoma, urinary tract, prostate, thyroid
	Amplifications	Lung (squamous cell), lung (adenocarcinoma), lung (small cell), lung (non-small cell), cervical, breast, head and neck, gastric, thyroid, oesophageal, endometrial, ovarian, glioblastoma
PIK3CB (phosphoinositide-3-kinase, catalytic, *β*-polypeptide)	Amplifications	Ovarian, breast
	Increase in activity and expression	Colon, bladder
PDPK1 (3-phosphoinositide dependent protein kinase-1)	Amplifications and overexpression	Breast
AKT (v-akt murine thymoma viral oncogene homologue)	AKT homologue 1 mutation (E17K) or amplifications	Breast, colon, ovarian, lung, gastric
	AKT homologue 2 amplifications	Ovarian, pancreas, head and neck, breast
	AKT homologue 3 mutation (E17K) or amplifications	Skin, glioblastoma
PIK3R1 (phosphoinositide-3-kinase, regulatory subunit-1)	Mutations	Glioblastoma, ovarian, colon
PTEN (phosphatase and tensin homologue)	Loss of heterozygosity	Gastric, breast, melanoma, prostate, glioblastoma
	Mutations	Endometrial, brain, skin, prostate, colon, ovary, breast, haematopoietic and lymphoid tissue, stomach, liver, kidney, vulva, urinary tract, thyroid, lung

A plethora of Akt inhibitors is under pre-clinical and clinical trials. Various drug trials have been initiated for Akt Inhibitors. However, miltefosine, is the only Akt inhibitor which has recently got approved by FDA albeit for use in treatment of leishmaniasis. Akt inhibitors can be divided into six major classes based on their mechanisms of action. The first class contains ATP competitive inhibitors of Akt. These include compounds such as CCT128930 and GDC-0068, which inhibit Akt2 and Akt1, respectively [26,27]. The latter has successfully entered Phase II trials. This category also includes the pan-Akt kinase inhibitors such as GSK2110183 (afuresertib), which has entered Phase II trials, GSK690693, which entered Phase I trials for refractory hematologic malignancies, but was withdrawn prior to enrolment, and AT7867, which is still in pre-clinical phase [28-30]. The second class contains lipid-based Akt inhibitors which act by preventing the generation of PIP3 by PI3K. This mechanism is utilized by phosphatidylinositol analogs such as Calbiochem Akt Inhibitors I, II and III (category no. 124005, 124008, 124009 respectively) or other PI3K inhibitors such as PX-866 [31]. This category also includes compounds such as Perifosine that entered phase III trials for colorectal cancer and multiple myeloma, but failed in 2012 [32]. The third class contains a group of compounds called psuedosubstrate inhibitors. These include compounds such as AKTide-2 T and FOXO3 hybrid [33,34]. The fourth class consists of allosteric inhibitors of AKT kinase domain, and include compounds such as MK2206. The fifth class consists of antibodies and include entities such as GST-anti-Akt1-MTS. The last class comprises of compounds that interact with the PH domain of Akt, and include compounds such as Triciribine and PX-316. Apart from these, other molecules such as the Akt-1 Antisense Oligonucleotide RX-0201, have also yielded favorable results [35].

In short, the use of Akt inhibitors as monotherapy or in combination with other anticancer drugs could be useful for combating drug resistance and improving response. Thus, comprehensive understanding of Akt and its linked signaling pathways (PI3K, PKB, mTOR etc.) is necessary to lead to newer drug development and use [36].

## Competing interests

The authors declare that that have no competing interests.

## Authors’ contributions

AS conceived the topic. JB was involved in drafting the initial manuscript. AS and AKN were involved in critically revising the manuscript, listed in decreasing order of their contributions. The authors have read and approved the manuscript. The authors did not receive any financial support/grant.

## References

[B1] Garofalo RS Orena SJ Rafidi K Torchia AJ Stock JL Hildebrandt AL Coskran T Black SC Brees DJ Wicks JR Severe diabetes, age-dependent loss of adipose tissue, and mild growth deficiency in mice lacking Akt2/PKB beta J Clin Invest 2003 8 2 197 208 12843127 10.1172/JCI16885 PMC164287

[B2] Yang ZZ Tschopp O Baudry A Dummler B Hynx D Hemmings BA Physiological functions of protein kinase B/Akt Biochem Soc Trans 2004 8 Pt 2 350 354 15046607 10.1042/bst0320350

[B3] Manning BD Cantley LC AKT/PKB signaling: navigating downstream Cell 2007 8 7 1261 1274 10.1016/j.cell.2007.06.009 17604717 PMC2756685

[B4] Pommery N Henichart JP Involvement of PI3K/Akt pathway in prostate cancer–potential strategies for developing targeted therapies Mini Rev Med Chem 2005 8 12 1125 1132 10.2174/138955705774933356 16375758

[B5] Song G Ouyang G Bao S The activation of Akt/PKB signaling pathway and cell survival J Cell Mol Med 2005 8 1 59 71 10.1111/j.1582-4934.2005.tb00337.x 15784165 PMC6741304

[B6] Whang YE Yuan XJ Liu Y Majumder S Lewis TD Regulation of sensitivity to TRAIL by the PTEN tumor suppressor Vitam Horm 2004 8 409 426 15110188 10.1016/S0083-6729(04)67021-X

[B7] Liang J Slingerland JM Multiple roles of the PI3K/PKB (Akt) pathway in cell cycle progression Cell Cycle 2003 8 2 339 345 12851486

[B8] Majumder PK Febbo PG Bikoff R Berger R Xue Q McMahon LM Manola J Brugarolas J McDonnell TJ Golub TR mTOR inhibition reverses Akt-dependent prostate intraepithelial neoplasia through regulation of apoptotic and HIF-1-dependent pathways Nat Med 2004 8 6 594 601 10.1038/nm1052 15156201

[B9] Somanath PR Razorenova OV Chen J Byzova TV Akt1 in endothelial cell and angiogenesis Cell Cycle 2006 8 5 512 518 10.4161/cc.5.5.2538 16552185 PMC1569947

[B10] Shiojima I Walsh K Role of Akt signaling in vascular homeostasis and angiogenesis Circ Res 2002 8 12 1243 1250 10.1161/01.RES.0000022200.71892.9F 12089061

[B11] Lefranc F Brotchi J Kiss R Possible future issues in the treatment of glioblastomas: special emphasis on cell migration and the resistance of migrating glioblastoma cells to apoptosis J Clin Oncol 2005 8 10 2411 2422 10.1200/JCO.2005.03.089 15800333

[B12] Thant AA Nawa A Kikkawa F Ichigotani Y Zhang Y Sein TT Amin AR Hamaguchi M Fibronectin activates matrix metalloproteinase-9 secretion via the MEK1-MAPK and the PI3K-Akt pathways in ovarian cancer cells Clin Exp Metastasis 2000 8 5 423 428 10.1023/A:1010921730952 11467775

[B13] Liu JP Studies of the molecular mechanisms in the regulation of telomerase activity FASEB J 1999 8 15 2091 2104 10593857 10.1096/fasebj.13.15.2091

[B14] Hsieh AC Truitt ML Ruggero D Oncogenic AKTivation of translation as a therapeutic target Br J Cancer 2011 8 3 329 336 10.1038/bjc.2011.241 21772331 PMC3172900

[B15] Cheng JQ Godwin AK Bellacosa A Taguchi T Franke TF Hamilton TC Tsichlis PN Testa JR AKT2, a putative oncogene encoding a member of a subfamily of protein-serine/threonine kinases, is amplified in human ovarian carcinomas Proc Natl Acad Sci U S A 1992 8 19 9267 9271 10.1073/pnas.89.19.9267 1409633 PMC50107

[B16] Cheng JQ Ruggeri B Klein WM Sonoda G Altomare DA Watson DK Testa JR Amplification of AKT2 in human pancreatic cells and inhibition of AKT2 expression and tumorigenicity by antisense RNA Proc Natl Acad Sci USA 1996 8 8 3636 3641 10.1073/pnas.93.8.3636 8622988 PMC39663

[B17] Xu X Sakon M Nagano H Hiraoka N Yamamoto H Hayashi N Dono K Nakamori S Umeshita K Ito Y Akt2 expression correlates with prognosis of human hepatocellular carcinoma Oncol Rep 2004 8 1 25 32 14654898

[B18] Agarwal E Brattain MG Chowdhury S Cell survival and metastasis regulation by Akt signaling in colorectal cancer Oncol Rep 2013 8 8 1711 1719 10.1016/j.cellsig.2013.03.025 PMC3686084 23603750

[B19] Soung YH Lee JW Nam SW Lee JY Yoo NJ Lee SH Mutational analysis of AKT1, AKT2 and AKT3 genes in common human carcinomas Oncology 2006 8 4 285 289 10.1159/000096289 17047397

[B20] Staal SP Molecular cloning of the akt oncogene and its human homologues AKT1 and AKT2: amplification of AKT1 in a primary human gastric adenocarcinoma Proc Natl Acad Sci U S A 1987 8 14 5034 5037 10.1073/pnas.84.14.5034 3037531 PMC305241

[B21] Knobbe CB Reifenberger G Genetic alterations and aberrant expression of genes related to the phosphatidyl-inositol-3′-kinase/protein kinase B (Akt) signal transduction pathway in glioblastomas Brain Pathol 2003 8 4 507 518 14655756 10.1111/j.1750-3639.2003.tb00481.x PMC8095764

[B22] Nakatani K Thompson DA Barthel A Sakaue H Liu W Weigel RJ Roth RA Up-regulation of Akt3 in estrogen receptor-deficient breast cancers and androgen-independent prostate cancer lines J Biol Chem 1999 8 31 21528 21532 10.1074/jbc.274.31.21528 10419456

[B23] Bellacosa A de Feo D Godwin AK Bell DW Cheng JQ Altomare DA Wan M Dubeau L Scambia G Masciullo V Molecular alterations of the AKT2 oncogene in ovarian and breast carcinomas Int J Cancer 1995 8 4 280 285 10.1002/ijc.2910640412 7657393

[B24] Lindhurst MJ Sapp JC Teer JK Johnston JJ Finn EM Peters K Turner J Cannons JL Bick D Blakemore L A mosaic activating mutation in AKT1 associated with the Proteus syndrome N Engl J Med 2011 8 7 611 619 10.1056/NEJMoa1104017 21793738 PMC3170413

[B25] Gupta AK McKenna WG Weber CN Feldman MD Goldsmith JD Mick R Machtay M Rosenthal DI Bakanauskas VJ Cerniglia GJ Local recurrence in head and neck cancer: relationship to radiation resistance and signal transduction Clin Cancer Res 2002 8 3 885 892 11895923

[B26] Yap TA Walton MI Hunter LJ Valenti M de Haven Brandon A Eve PD Ruddle R Heaton SP Henley A Pickard L Preclinical pharmacology, antitumor activity, and development of pharmacodynamic markers for the novel, potent AKT inhibitor CCT128930 Mol Cancer Ther 2011 8 2 360 371 10.1158/1535-7163.MCT-10-0760 21191045 PMC4944842

[B27] Blake JF Xu R Bencsik JR Xiao D Kallan NC Schlachter S Mitchell IS Spencer KL Banka AL Wallace EM Discovery and preclinical pharmacology of a selective ATP-competitive Akt inhibitor (GDC-0068) for the treatment of human tumors J Med Chem 2012 8 18 8110 8127 10.1021/jm301024w 22934575

[B28] ClinicalTrials.gov | An Open-Label Phase 2 Study of Ofatumumab (Arzerra) in Combination With Oral GSK2110183 in the Treatment of Relapsed and Refractory Chronic Lymphocytic Leukemia (CLL) [ http://clinicaltrials.gov/ct2/show/NCT01532700]

[B29] ClinicalTrials.gov | Study to Investigate AKT Inhibitor GSK690693 in Subjects With Relapsed or Refractory Hematologic Malignancies [ http://clinicaltrials.gov/ct2/show/NCT00666081]

[B30] Grimshaw KM Hunter LJ Yap TA Heaton SP Walton MI Woodhead SJ Fazal L Reule M Davies TG Seavers LC AT7867 is a potent and oral inhibitor of AKT and p70 S6 kinase that induces pharmacodynamic changes and inhibits human tumor xenograft growth Mol Cancer Ther 2010 8 5 1100 1110 10.1158/1535-7163.MCT-09-0986 20423992 PMC4825853

[B31] Hu Y Qiao L Wang S Rong SB Meuillet EJ Berggren M Gallegos A Powis G Kozikowski AP 3-(Hydroxymethyl)-bearing phosphatidylinositol ether lipid analogues and carbonate surrogates block PI3-K, Akt, and cancer cell growth J Med Chem 2000 8 16 3045 3051 10.1021/jm000117y 10956212

[B32] Kondapaka SB Singh SS Dasmahapatra GP Sausville EA Roy KK Perifosine, a novel alkylphospholipid, inhibits protein kinase B activation Mol Cancer Ther 2003 8 11 1093 1103 14617782

[B33] Luo Y Smith RA Guan R Liu X Klinghofer V Shen J Hutchins C Richardson P Holzman T Rosenberg SH Pseudosubstrate peptides inhibit Akt and induce cell growth inhibition Biochemistry 2004 8 5 1254 1263 10.1021/bi034515p 14756561

[B34] Barnett SF Bilodeau MT Lindsley CW The Akt/PKB family of protein kinases: a review of small molecule inhibitors and progress towards target validation Curr Top Med Chem 2005 8 2 109 125 10.2174/1568026053507714 15853641

[B35] National Cancer Institute, NIH. Drug Dictionary [ http://www.cancer.gov/drugdictionary]

[B36] Pal SK Reckamp K Yu H Figlin RA Akt inhibitors in clinical development for the treatment of cancer Expert Opin Investig Drugs 2010 8 11 1355 1366 10.1517/13543784.2010.520701 20846000 PMC3244346

